# A Strategy to Optimize the Generation of Stable Chromobody Cell Lines for Visualization and Quantification of Endogenous Proteins in Living Cells

**DOI:** 10.3390/antib8010010

**Published:** 2019-01-10

**Authors:** Bettina-Maria Keller, Julia Maier, Melissa Weldle, Soeren Segan, Bjoern Traenkle, Ulrich Rothbauer

**Affiliations:** 1 Pharmaceutical Biotechnology, Eberhard Karls University, 72076 Tuebingen, Germany; Bettina.Keller@nmi.de (B.-M.K.); J.Maier@med.uni-tuebingen.de (J.M.); melissa.weldle@student.uni-tuebingen.de (M.W.); Bjoern.Traenkle@nmi.de (B.T.); 2 Natural and Medical Sciences Institute at the University of Tuebingen, Markwiesenstr. 55, 72770 Reutlingen, Germany; Soeren.Segan@nmi.de

**Keywords:** nanobodies, chromobodies, live-cell imaging, compound screening, cellular models

## Abstract

Single-domain antibodies have emerged as highly versatile nanoprobes for advanced cellular imaging. For real-time visualization of endogenous antigens, fluorescently labelled nanobodies (chromobodies, CBs) are introduced as DNA-encoded expression constructs in living cells. Commonly, CB expression is driven from strong, constitutively active promoters. However, high expression levels are sometimes accompanied by misfolding and aggregation of those intracellular nanoprobes. Moreover, stable cell lines derived from random genomic insertion of CB-encoding transgenes bear the risk of disturbed cellular processes and inhomogeneous CB signal intensities due to gene positioning effects and epigenetic silencing. In this study we propose a strategy to generate optimized CB expressing cell lines. We demonstrate that expression as ubiquitin fusion increases the fraction of intracellularly functional CBs and identified the elongation factor 1α (EF1-α) promoter as highly suited for constitutive CB expression upon long-term cell line cultivation. Finally, we applied a CRISPR/Cas9-based gene editing approach for targeted insertion of CB expression constructs into the adeno-associated virus integration site 1 (AAVS1) safe harbour locus of human cells. Our results indicate that this combinatorial approach facilitates the generation of fully functional and stable CB cell lines for quantitative live-cell imaging of endogenous antigens.

## 1. Introduction

Cell culture models provide substantial information on various cellular responses ranging from exposure to small chemical compounds to genetically mediated target depletion [1]. In combination with advanced microscopy techniques such as quantitative live-cell imaging they can be directly employed to monitor cellular events and phenotypic changes with spatial and temporal resolution. In vitro cell models are essential tools in basic biomedical research and are applied to identify novel cellular targets and compound candidates in pharmaceutical development [2,3]. For imaging-based analysis, expression of fluorescent fusion proteins (FP fusions) is the most commonly used approach to study localization and dynamic changes of proteins in living cells. Commercially available FP-based live-cell assays enable the investigation of processes such as cell proliferation, apoptosis or DNA damage. However, this approach is limited to the visualization of ectopically expressed FP fusions, which may considerably differ from their endogenous counterparts in terms of expression level, activity, localization and protein half-life [4,5,6,7,8]. Recently evolved gene editing methods such as the CRISPR/Cas9 or the ZFN/TALEN technology can now be used to generate cell lines expressing fluorescently tagged proteins under their endogenous expression control. While these technologies offer novel opportunities for protein analysis the possibility of functional interference by the attached fluorescent moiety still remains.

Intracellular affinity reagents provide a straightforward approach to overcome the drawbacks of FP fusions as they exclusively visualize and trace the dynamics of endogenous target structures [9,10]. Due to their robustness and structural simplicity, fluorescently labelled nanoprobes derived from single-domain antibody fragments of camelids (chromobodies, CBs) can be selected to detect antigens in their native surroundings in living cells [9,11]. Since their first description in 2006, numerous CBs have been established and applied to visualize and monitor their target molecules in various cell models and whole organisms [12,13,14,15,16,17].

**Figure 1:**
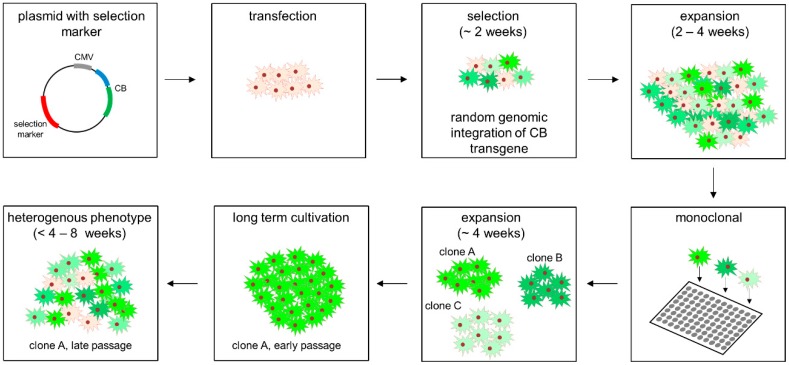
Typical workflow of stable chromobody cell line generation following random genomic integration of chromobody (CB) transgene.

While transient expression of CBs is sufficient to visualize the dynamics and relocalization of endogenous proteins on single-cell level, larger screening campaigns require the development of stable cell lines with a homogenous and consistent CB expression [16,17,18]. Although various stable CB cell lines have been reported by us and others, the selection and characterization of cell lines compatible for quantitative live-cell imaging is still very cumbersome. Most importantly, quantitative image analysis often suffers from inconsistent CB expression levels, aggregation and a strong cell-to-cell variance of CB fluorescence intensities (Figure 1). In this context it has to be considered that in most available cell models CB expression is driven from the strong and constitutively active cytomegalovirus-(CMV) promoter. Although high CB levels are desired in general, elevated transgene expression is sometimes accompanied by misfolding and aggregation [19,20]. Additionally, following the conventional workflow of stable cell line generation CB transgenes are usually randomly inserted in the cellular genome (Figure 1). Notably, this not only bears the risk of unintended genomic manipulation which might affect cellular processes but can also lead to inconsistent CB signal intensities due to the integration of different copy numbers of the CB transgene, chromatin positioning effects [21] and epigenetic silencing of the promoter upon continuous sub-culturing [22].

Here, we explore a combination of previously established methods to improve the generation of stable CB cell lines. Based on comparative analyses we propose to (i) implement our recently developed turnover-accelerated CBs expressed as ubiquitin fusions in order to monitor changes in the antigen concentration and to avoid intracellular CB aggregation, (ii) select a promoter, which is less prone to epigenetic silencing and (iii) insert these optimized CB expression constructs into the adeno-associated virus integration site 1 (AAVS1) safe harbour locus of human cells using a targeted CRISPR/Cas9 gene editing approach.

## 2. Material and Methods

### 2.1. Expression Constructs

All primer sequences, synthesized DNA fragments and plasmids are listed in Table S1. All expression constructs used in this study are listed in Table S2.

The expression constructs encoding for Ub-M-LMN-CB and Ub-R-LMN-CB were generated by amplification the Lamin-NB from the Lamin-CB plasmid [23] using the following primer set: NB-ubi-for and NB-ubi-rev. The amplified DNA fragment was purified and ligated into PstI and BspEI restriction site of Ub-M-BC1-eGFP and Ub-R-BC1-eGFP (both [24]), respectively. To generate the Ub-R-BC1-CB containing the EF1-α promoter (referred to as EF1-α_Ub-R-BC1-eGFP), the EF1-α promoter was synthesized as gBlock^®^ gene fragment (IDT, integrated DNA technologies) and Gibson Assembly [25] was performed after restriction digest of Ub-R-BC1-eGFP [24] using the restriction enzymes AseI und NheI. Fragments were assembled using Gibson-Assembly Master Mix (New England Biolabs, Ipswich, MA, USA) according to the manufacturer’s protocol. The BC1-CB expression construct containing the human β-actin promoter (referred to as h-βact_Ub-R-BC1-eGFP) was generated by amplification of the promoter from pDRIVE-hβ-Actin plasmid ([26], kindly provided by Hiroyuki Konishi, Aichi Medical University School of Medicine, Japan) using the primer set β-actin-promoter-for and β-actin-promoter-rev and ligation into AseI und NheI digested Ub-R-BC1-eGFP [24]. To eliminate the PstI restriction site within the hβ-act promoter site-directed mutagenesis was performed utilizing the primer set β-actin-promoter-mutPstI-for and β-actin-promoter-mutPstI-rev using the Q5 Site-Directed Mutagenesis Kit (New England Biolabs) according to the manufacturer’s protocol. For molecular cloning of the AAVS1-CB-donor plasmid (as described in Figure 4A) two DNA fragments were used, which were produced by gene synthesis. At first, AAVS1-CB-donor fragment 1 (gene synthesis, plasmid DNA, IDT) was digested using PciI and MfeI and directly ligated into a PciI and MfeI digested pEGFP-N1backbone (Clontech, Mountain View, CA, USA). Secondly, the resulting plasmid was digested with MfeI and XbaI and completed by AAVS1-CB-donor fragment 2 insertion (gBlock^®^ gene fragment), which was amplified with the following primer set: AAVS1-CB-donor-fragment-2-for and AAVS1-CB-donor-fragment-2-rev. In a last step the complete cassette was sequence verified using primers Seq-AAVS1-CB-donor-1 - 4 for sequencing. To generate an AAVS1-CB-donor plasmid containing the EF1-α_Ub-R-BC1-CB (resulting plasmid AAVS1_Ub-R-BC1-CB), we used the AseI and XbaI restriction site to integrate the CB cassette including the promoter. For the AAVS1-CB-donor plasmid containing the EF1-α_Ub-R-ACT-CB (AAVS1_Ub-R-ACT-CB) the actin NB was amplified with the primer set NB-ubi-for and NB-ubi-rev and ligated into AAVS1_Ub-R-BC1-CB backbone using PstI and BspEI restriction site. All generated constructs in this study were sequence analysed after cloning.

### 2.2. Cell Culture, Transfection, Stable Cell Line Generation and Compound Treatment

HeLa Kyoto cells (Cellosaurus no. CVCL_1922) were obtained from S. Narumiya (Kyoto University, Japan), whereas DLD-1 (ATCC^®^ Number CCL-221^TM^) and HCT116 (ATCC^®^ Number CCL-247^TM^) were obtained from ATCC. All cell lines were tested negative for mycoplasma using the PCR mycoplasma kit Venor GeM Classic (Minerva Biolabs, Berlin, Germany) and the Taq DNA polymerase (Minerva Biolabs). Since this study does not include cell line-specific analysis, all cell lines were used without additional authentication. All cell lines were maintained according to standard protocols. Briefly, growth media containing DMEM (high glucose, pyruvate, ThermoFisher Scientific, Waltham, MA, USA) for HeLa Kyoto and HCT116 cells and RPMI 1640 (ThermoFisher Scientific) for DLD-1 cells supplemented with 10% (*v*/*v*) foetal bovine serum (FCS, ThermoFisher Scientific) and penicillin-streptomycin (ThermoFisher Scientific) were used for cultivation. Cells were routinely passaged using 0.05% trypsin-EDTA (ThermoFisher Scientific) and were cultivated at 37 °C in a humidified chamber with a 5% CO_2_ atmosphere. Plasmid DNA was transfected with Lipofectamine 2000 (ThermoFisher Scientific) in HeLa cells, whereas DLD-1 and HCT116 were transfected with TransIT-X2^®^ (Mirus Bio, Madison, WI, USA) according to the manufacturer’s instructions. For site-directed integration of the CB into AAVS1 genomic locus, 5 × 10^5^ cells were co-transfected with 2.5 µg of the respective donor plasmid and 2.5 µg plasmid encoding for Cas9 nuclease and gRNA specific for the AAVS1 locus. 24 h post transient transfection cells were subjected to a 48 h selection period using 0.6 µg/mL puromycin dihydrochloride (Sigma-Aldrich, St. Louis, MO, USA). Puromycin-resistant cells were allowed to grow for one week before single clones were isolated. Single clones were analysed regarding the CB expression level by fluorescence microscopy. To verify site-directed integration of the CB-donor plasmid at the AAVS1 locus, genomic DNA of the single clones and the respective parental cell line was isolated using QIAamp DNA mini Kit (QIAGEN, Venlo, the Netherlands). Next, the primer pair genPCR-AAVS1-int-for and genPCR-AAVS1-int-rev [27] was used for PCR-based genotyping. CB integration into the AAVS1 locus results in an amplicon of ~1400 bps (strategy outlined in Figure 4B). The resulting amplicon was purified and verified via sequence analysis. Compound treatment with FH535 (Sigma-Aldrich) and XAV939 (MedChemExpress) was performed for up to 24 h. For dose-response experiments cells were treated with 1 µM, 10 µM and 50 µM for FH535 and 1 µM, 5 µM and 10 µM for XAV939.

### 2.3. Fluorescence Imaging, Image Segmentation and Analysis

For fluorescence imaging 5000 cells per well were plated in a black µClear 96-well plate (Greiner Bio-One, Kremsmünster, Austria). For staining of the nuclei in PFA-fixed cells 0.02 µg/mL 4′,6-diamidino-2-phenylindole (DAPI, Sigma-Aldrich) was used while living cells were continuously incubated with 2 µg/mL Hoechst33258 (Sigma). Images were acquired with an ImageXpress micro XL system (Molecular Devices, San Jose, CA, USA) and analysed by MetaXpress software (64 bit, 6.2.3.733, Molecular Devices). Fluorescence images comprising a statistically relevant number of cells (>200) were acquired for each condition. For quantitative fluorescence analysis the mean fluorescence of the respective CB expression construct in mCherry or mCherry-CTNNB1 transfected cells was determined. Using the Custom Module Editor (version 2.5.13.3) of the MetaXpress software, we established an image segmentation algorithm that identifies areas of interest based on the parameters of size, shape and fluorescence intensity above local background. To segment the whole cell including the nucleus and the cytosolic compartment, the ectopically antigen mCherry-CTNNB1 or its respective control mCherry was used to generate the corresponding segmentation mask. The average fluorescence intensities were determined for each image followed by subtraction of background fluorescence. From these values the mean fluorescence and standard errors were calculated for three independent biological replicates and student’s *t*-test was used for statistical analysis.

### 2.4. Western Blot

DLD-1_AAVS1_Ub-R-BC1-CB cells were seeded in a 10 cm² cell culture dish (Corning) with 3 × 10^6^ cells per dish. After two days the cells were treated with DMSO or 10 µM FH535 for 24 h. For the lysis cells were harvested with a cell scraper and cold PBS and centrifuged at 500× *g* and 4 °C for 3 min. Per 50 µL pellet 100 µL lysis buffer (10 mM Tris/HCl, pH 7.5, 150 mM NaCl, 0.5 mM EDTA, 0.5% NP40, 1mM PMSF, 1× protease inhibitor cocktail (Serva, Heidelberg, Germany), 1× phosphatase inhibitor (PhosSTOP, Roche, Basel, Switzerland) 250 µg/µL DNase, 2.5 mM MgCl_2_) was added. The samples were pipetted 30 times every 10 min for 30 min and centrifuged at 16,000× *g* for 10 min at 4 °C. The samples were boiled in 2× reducing SDS-sample buffer (60 mM Tris/HCl, pH 6.8, 2% (*w*/*v*) SDS, 5% (*v*/*v*) 2-mercaptoethanol, 10% (*v*/*v*) glycerol, 0.02% bromophenol blue) for 15 min at 95 °C. The SDS-PAGE and western blot were performed according to standard procedure. For western blotting the proteins were transferred on nitrocellulose membrane (Amersham, GE Healthcare, Chicago, IL, USA). The blots were scanned on a Typhoon-Trio laser scanner (GE Healthcare). The analysis was done with Image Quant TL Toolbox (GE Healthcare, version 7.0).

For immunoblot detection following antibodies were used: Total-CTNNB1 (mouse monoclonal, BD, #610154, 1:1000), Active-CTNNB1 (mouse monoclonal, 8E7, Millipore, #05-665, 1:500), Tubulin (mouse monoclonal, Sigma-Aldrich, #T9026, 1:2000). For the detection of the primary antibodies fluorescently labelled secondary antibodies (goat-anti-mouse, Alexa Fluor 647, ThermoFisher Scientific, 1:1000) was used.

## 3. Results

### 3.1. Expression of CBs as Ubiquitin Fusions Reduces Intracellular Aggregation

Recently, we observed that CBs are stabilized in the presence of their antigens and conceived a strategy to reduce the levels of non-bound CBs [24]. We utilized the N-end rule describing the N-terminal amino acid as one of the key determinants for the half-life of proteins [28,29]. Based on the ubiquitin fusion technique, we designed novel CB expression constructs comprising a N-terminal ubiquitin fusion, which is co-translationally cleaved by deubiquitinases. By this technique we were able to generate CBs displaying any desired amino acid at their N-termini and identified that arginine (Arg, R) mediated the fastest CB turnover when exposed at the N-terminus. Functionally, we showed that CBs containing a N-terminal Arg were highly antigen responsive and suitable to monitor and quantify dynamic changes of the concentration of endogenous antigens in real-time [24]. Besides the generation of proteins with a desired N-terminus, the ubiquitin fusion technique has also been reported to increase solubility and functionality of ectopically expressed proteins [30]. According to these findings we noticed that ubiquitin fusions of CBs are less prone to aggregation irrespective of the adjacent amino acid. Thus we asked, whether these potential benefits can be transferred to CBs such as the lamin-specific CB (LMN-CB), which has previously been shown to form aggregates upon high expression levels in HeLa cells [23,31].

**Figure 2:**
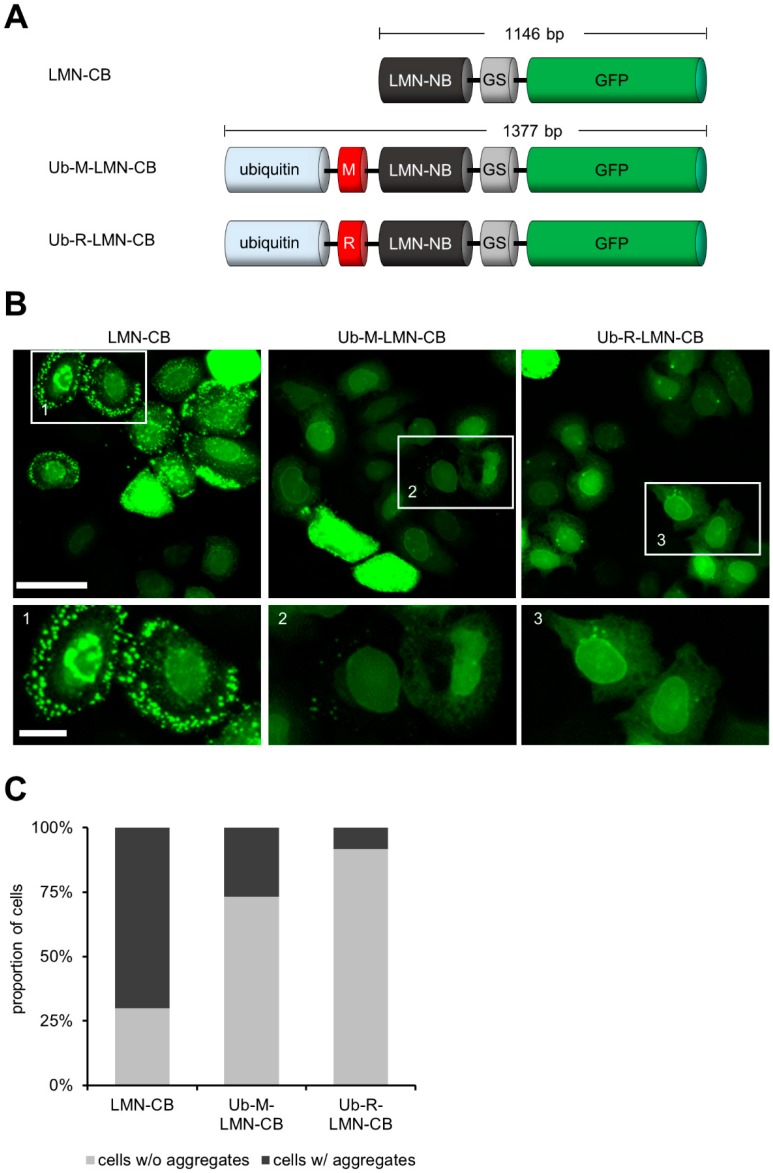
N-terminal fusion to ubiquitin reduces aggregation of the LMN-CB upon expression in HeLa cells. (**A**) Schematic illustration of different expression constructs: one encoding for an unmodified LMN-CB and two constructs encoding the CB as ubiquitin fusion displaying either methionine (Ub-M-LMN-CB) or arginine (Ub-R-LMN-CB) as the N-terminal amino acid. (**B**) Representative fluorescence images of HeLa cells transiently expressing either LMN-CB, Ub-M-LMN-CB or Ub-R-LMN-CB. Fluorescence images were acquired 24 h post transfection, scale bars images: 50 µm, insets: 10 µM. (**C**) Quantification of HeLa cells showing aggregates upon transient expression of the indicated LMN-CB variants. Number of analysed cells: LMN-CB: 198; Ub-M-LMN-CB: 142; Ub-R-LMN-CB: 155.

To test whether the ubiquitin fusion approach is able to reduce the amount of aggregated LMN-CB we generated expression constructs encoding for Ub-M-LMN-CB and the turnover-accelerated version Ub-R-LMN-CB (Figure 2A). For microscopic analysis HeLa cells were transiently transfected either with the modified LMN-CB constructs or the original LMN-CB expression plasmid. 24 h post transfection HeLa cells were subjected to fluorescence imaging and the percentage of cells containing aggregates were determined for each LMN-CB variant (Figure 2B). In most of the cells expressing the original LMN-CB construct we observed the formation of fluorescent aggregates (~70% of all analysed cells). Upon transient expression of the LMN-CB as an ubiquitin fusion followed by a methionine (Ub-M-LMN-CB) the number of cells displaying aggregates was significantly reduced to ~27% and to only 8% in cells transfected with the turnover-accelerated version of the LMN-CB (Ub-R-LMN-CB) (Figure 2C). Notably, while the nuclear lamina was hardly detectable in the majority of cells expressing the original LMN-CB construct, the modified CB versions displayed the typical nuclear rim structure more prominently. These results confirmed our previous observations showing that the addition of the ubiquitin moiety increased the intracellular solubility of CBs while leaving the binding properties unaffected (Figure 2B).

### 3.2. Comparison of CB Expression Driven by the CMV-, EF1-α or β-actin Promoter to Monitor Changes in Antigen Concentration

As recently described, high expression levels of turnover-accelerated CBs are necessary to optically monitor rapid changes in antigen concentration within living cells via antigen-mediated CB stabilization (AMCBS) [24]. For larger screening campaigns using quantitative live-cell imaging the expression levels have to be consistently high over longer cultivation periods. One major critical factor causing reduction and thus heterogeneities in transgene expression lies within the epigenetic silencing of the promoter of the transgene, mainly caused by DNA methylation [32,33] and/or histone modification [34]. While the constitutive CMV promoter provides suitable expression levels, this viral promoter is reported to be highly sensitive to DNA methylation [35]. Indeed, after prolonged cultivation times we noticed heterogeneous CB fluorescence intensities in cell lines comprising a stable integration of the CB transgene controlled by the CMV promoter (Figure S1).

To optimize the stability of CB expression suitable for monitoring changes of endogenous antigen concentration we aimed for a strong promoter that is less prone to epigenetic silencing. Thus, we compared CMV-driven expression with the expression mediated by the human elongation factor 1α (EF1-α) promoter and the human β-actin (h-βact) promoter. While all three candidates were described to convey medium-to-high expression levels within different cell lines [26,36] both, the EF1-α and h-βact promoter were previously reported to be less prone to epigenetic silencing and maintain stable transgene expression levels over several passages [26,37].

**Figure 3:**
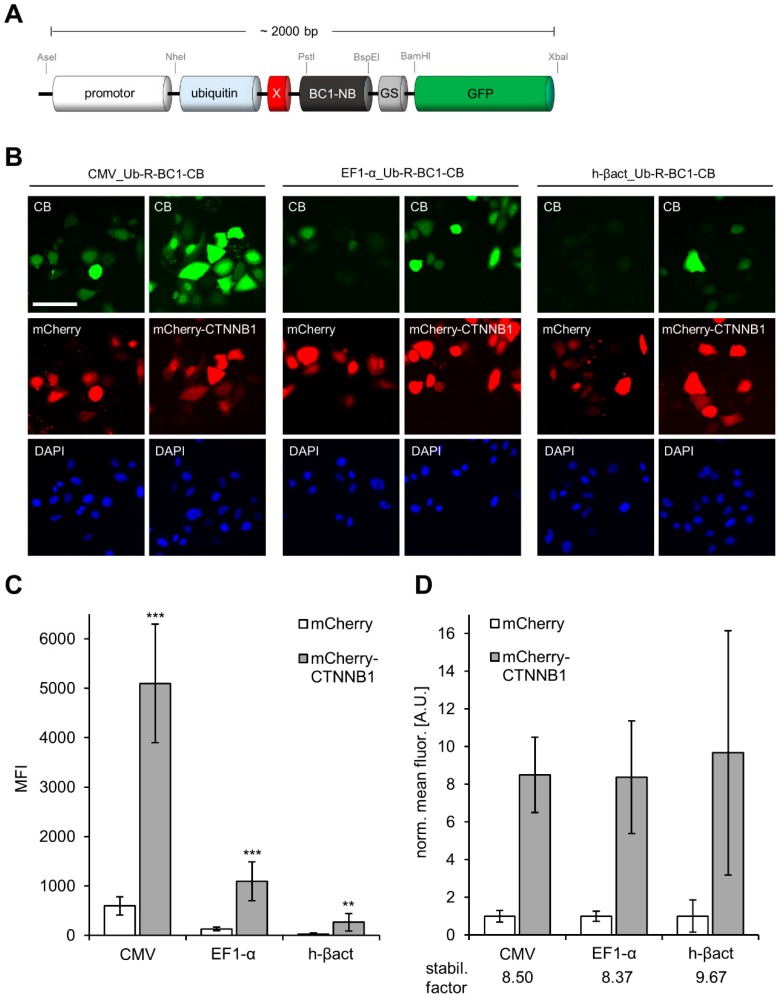
Quantitative image analysis of promoter-driven Ub-R-BC1-eGFP expression. (**A**) Schematic illustration of the CB expression construct for promoter testing. Promoter sequences can easily be exchanged using unique indicated restriction sites (AseI, NheI). (**B**) Representative images of HeLa cells transiently expressing Ub-R-BC1-CB driven by different promoters (CMV, EF1-α and h-βact) in combination with either mCherry (control) or mCherry-CTNNB1 (antigen). Nuclei were stained with DAPI, scale bar: 50 µm. (**C**) Bar chart represents mean CB fluorescence intensity (MFI) detected by quantitative fluorescence imaging in either control (mCherry) or antigen (mCherry-CTNNB1) expressing cells (*n* = 3, >200 cells each). (**D**) For every promoter construct, MFI of the CB in antigen expressing cells was normalized to the respective CB-signal determined in cells co-expressing mCherry as control, leading to the indicated stabilization factors. Error bars: S.D. Statistical analysis was performed using student’s *t*-test, *** *p* < 0.001, ** *p* < 0.01.

For comparative analysis we replaced the CMV promoter in our previously reported expression construct encoding the turnover-accelerated β-catenin (CTNNB1)-specific BC1-CB (Ub-R-BC1-CB, [24]) by the EF1-α or h-βact promoter (Figure 3A).

We compared the expression levels and the performance with regard to antigen-mediated stabilization of the original CMV-driven and the newly generated EF1-α or h-βact-driven CB constructs by transfecting HeLa cells either in combination with mCherry as control or mCherry-CTNNB1 as the corresponding antigen. Quantitative fluorescence imaging revealed substantial differences in CB expression levels (Figure 3B,C). For the CMV-driven expression we observed the highest expression levels within HeLa cells with a mean fluorescence intensity (MFI) of ~700 in mCherry-transfected control cells and a MFI of ~5000 in the presence of mCherry-CTNNB1. An intermediate strength in CB expression was determined for the EF1-α-containing variant indicated by a MFI of ~130 in control cells and a MFI of ~1000 in the presence of the antigen. For the h-βact-driven expression we detected rather weak signals, which were close to background level. Interestingly, similar stabilization factors (8.5–9.7) were calculated for all constructs, indicating that AMCBS was not affected by the exchange of the promoter (Figure 3C). Considering that EF1-α promoter is less sensitive to DNA methylation [37] but provides similar CB expression levels compared to the original CMV promoter, we decided to implement the EF1-α promoter in our strategy to generate optimized stable CB cell lines.

### 3.3. Design and Construction of AAVS1 Donor Vector for Site-Directed Stable Integration of Turnover-Accelerated CBs

Typically, the generation of stable CB cell models is based on the transfection of a cell line with a CB expression vector comprising a selection marker, which for example, confers resistance to antibiotics. Subsequently, cells are continuously cultivated in the presence of appropriate antibiotics to select clones that comprise a stable genomic integration of the CB transgene (Figure 1). Although this workflow was successfully applied to generate numerous stable CB cell lines, some pitfalls have to be considered. As the integration of the CB transgene occurs randomly, neither a prediction about the chromatin structure at the integration site can be made nor the number of CB transgene copies within the cellular host can be foreseen. Notably, the site of integration has a major effect on the expression levels of the transgene summarized as positioning effect [38]. Additionally, such stable cell lines have to be continuously cultured under constant selective pressure, which has been reported to affect host cell physiology, genetic stability and metabolism [39,40,41]. To address these shortcomings, we aimed to establish a new protocol that allows site-directed integration of turnover-accelerated CBs into the host cell DNA by applying the CRISPR/Cas9 gene editing technology.

**Figure 4:**
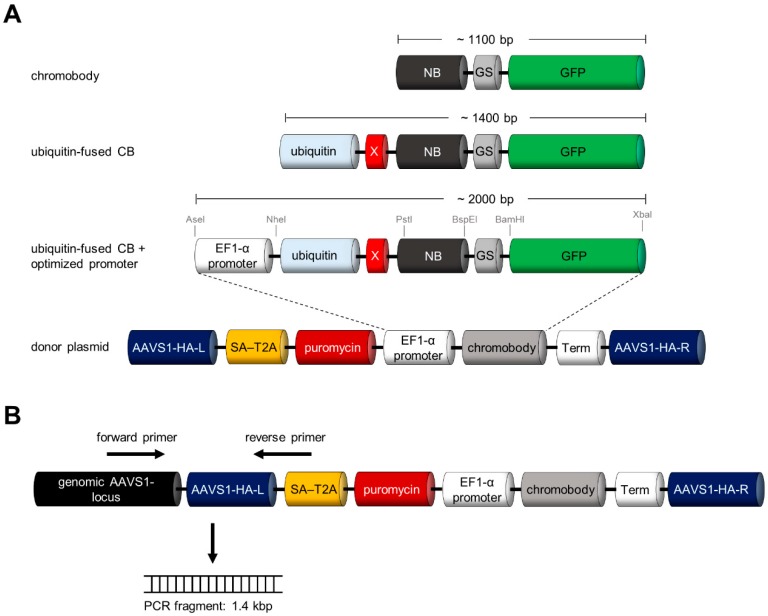
Strategy for stable integration of turnover-accelerated CBs into the human AAVS1 locus. (**A**) Schematic illustration of CB containing donor plasmid for stable integration into the human AAVS1 locus. (**B**) Strategy for PCR-based genotyping of host cell DNA to verify site-directed CB integration.

Recently, the adeno-associated virus site 1 (AAVS1, position 19q13.42), located in the first intron of the protein phosphatase 1 regulatory subunit 12C (PPP1R12C), was described as genomic safe-harbour (GSH) integration site [27,42,43,44,45,46]. As transgene expression from this GSH integration site was previously reported to result in robust and persistent protein levels [47], we chose the AAVS1 locus to integrate our turnover-accelerated CBs. For targeted engineering we constructed a donor plasmid containing a turnover-accelerated CB expression cassette, which is driven by an EF1-α promoter and flanked by AAVS1-specific homology arms (HA-L/R) (Figure 4A). Additionally, a puromycin resistance gene containing a splice acceptor site (SA) linked to a self-cleaving peptide sequence (T2A) was added. Upon correct genomic integration, the expression of the puromycin N-acetyl-transferase will be driven by the endogenous PPP1R12C promoter (Figure 4A), which allows the selection of clones that underwent the desired CRISPR event. All fragments within the construct were sequence-optimized and the indicated restriction sites (Figure 4A) allow an easy exchange of the different components including promoter, nanobody binding moiety and fluorescent marker. In addition, we adapted a PCR-based genotyping strategy to verify clones comprising a correct transgene integration (illustrated in Figure 4B [27]).

### 3.4. Site-Directed Integration of the Turnover-Accelerated Actin-Specific CB into the AAVS1 Locus

**Figure 5:**
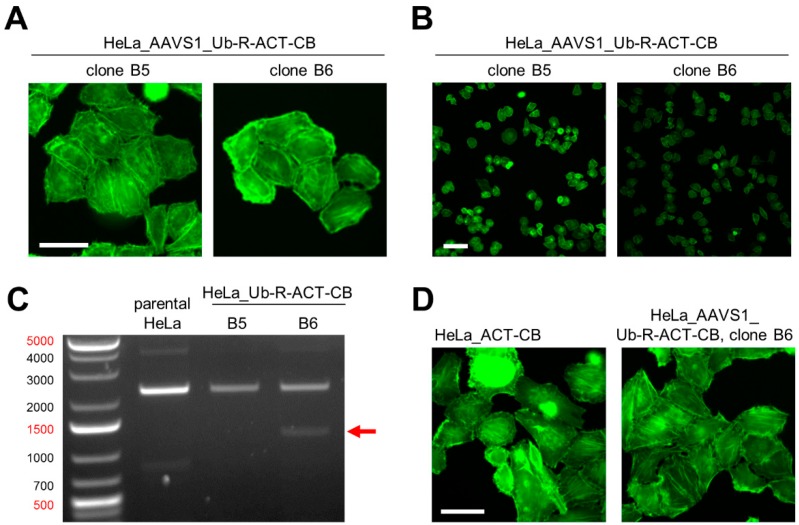
Generation of a HeLa cell line expressing Ub-R-ACT-CB stably integrated into the AAVS1 locus by using CRISPR/Cas9 technology. (**A**) Fluorescence images of two CRISPR-engineered HeLa cell clones expressing ACT-CB, scale bar: 50 µM. (**B**) Comparison of fluorescence intensity between HeLa_AAVS1_Ub-R-ACT-CB clone B5 and B6, scale bar: 100 µm. (**C**) PCR-based genotyping of HeLa_AAVS1_Ub-R-BC1-CB clones B5 and B6 in comparison to parental HeLa cells. Genomic DNA of the monoclonal cells was extracted and subjected to PCR using the genotyping strategy illustrated in Figure 4B. Shown are the resulting PCR products (indicated by arrow) on a 1% agarose gel stained with ethidiumbromid. (**D**) Representative fluorescence images of living HeLa cells stably expressing the respective ACT-CB. Left image illustrates HeLa_ACT-CB cells generated by random integration of the non-modified ACT-CB. The right image shows HeLa_AAVS1_Ub-R-ACT-CB cells generated by site-directed integration of the ubiquitin-modified ACT-CB using CRISPR/Cas9 technology, scale bar: 50 µm.

We applied this strategy to generate HeLa cells stably expressing the actin-specific CB (ACT-CB). The ACT-CB has been previously shown to bind to F-actin without affecting the dynamic reorganization of the cytoskeleton [15], thus the detection of filamentous actin provides a suitable read-out to validate the functionality of the CB transgene upon CRISPR/Cas9-mediated integration. In a first step, we co-transfected HeLa cells with plasmids encoding for (i) Cas9 nuclease and the respective gRNA for the AAVS1 locus [27] and (ii) the donor plasmid containing the turnover-accelerated ACT-CB (Ub-R-ACT-CB, Figure 4A). After transfection, cells were cultured for 48 h in the presence of puromycin to enrich cells that underwent stable AAVS1 integration of the CB transgene. After clonal expansion, we identified two clones (referred to as clone B5 and clone B6) showing filamentous structures indicative for the actin cytoskeleton (Figure 5A). Both clones were similar in cell morphology and size and displayed a homogenous expression of the ACT-CB. However, we noticed that the fluorescence intensity of clone B5 was slightly higher compared to clone B6 (Figure 5B). PCR-based genotyping (as outlined in Figure 4B) revealed for clone B6 an amplicon at the expected size of ~1400 bps (Figure 5C), indicating that a correct CB transgene integration at the AAVS1 locus was only obtained for clone B6, which was further verified by sequence analysis of the PCR fragment. Next, we qualitatively compared the morphology of the novel CRISPR-engineered HeLa_AAVS1_Ub-R-ACT-CB cell line with a stable ACT-CB expressing HeLa cell line previously generated by random integration of a CMV-driven, non-turnover-accelerated ACT-CB (Figure 5D). Fluorescence live-cell imaging revealed a more homogenous CB expression and cell morphology for the CRISPR-modified cell clone, while we detected a quite heterogeneous cell morphology along with some CB aggregates in cells stably expressing the non-modified ACT-CB transgene (Figure 5D).

### 3.5. Site-Directed Integration of the Turnover-Accelerated BC1-CB into the AAVS1 Locus of Human CRC Cell Lines

In a next step, we aimed to apply our approach to generate cell lines, which are suitable to monitor changes in endogenous protein concentration upon compound treatment by quantitative image analysis of the CB signal. In previous studies we demonstrated that our CTNNB1-specific CB (BC1-CB) traces changes in the levels of transcriptionally active, hypophosphorylated CTNNB1 upon induction of the β-catenin/WNT pathway [16,24]. These findings motivated us to generate more sophisticated CB cell models to monitor the effects of compounds on the reduction of endogenous CTNNB1 levels by following the BC1-CB signal. In over 90% of all colorectal cancer the β-catenin/WNT signalling pathway is mutated [48] resulting in an accumulation of transcriptionally active CTNNB1 involved in the initiation and progression of this cancer type. Consequently, cellular models which can be employed to screen for compound candidates affecting the endogenous levels of this particular CTNNB1 fraction would be advantageous for preclinical drug development. Thus, we chose two human colorectal carcinoma (CRC) cell lines DLD-1 and HCT116, which exhibit elevated level of active CTNNB1 to generate disease relevant CB-based screening cell lines [49].

**Figure 6:**
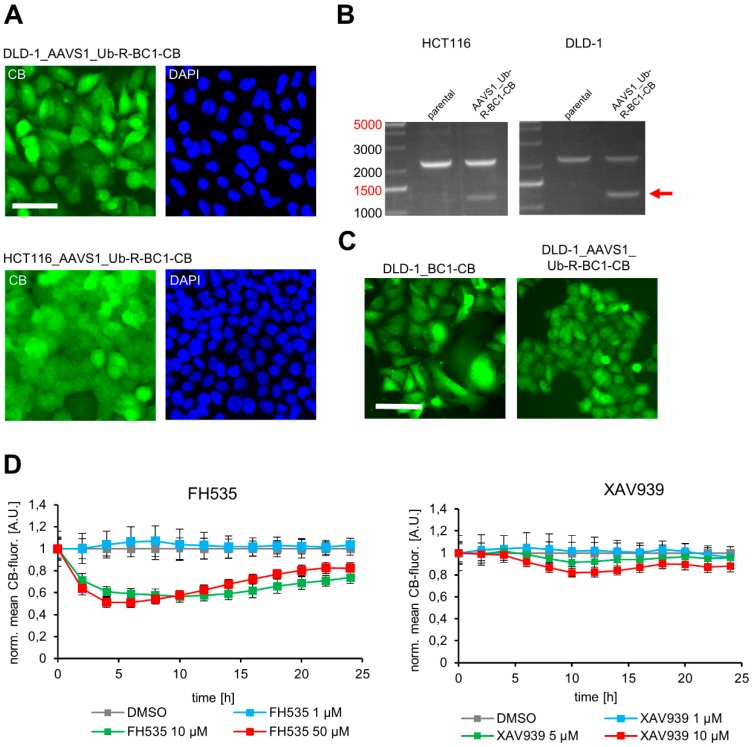
Generation of colorectal carcinoma cell lines with stable AAVS1 integration of the turnover-accelerated Ub-R-BC1-CB encoding transgene. (**A**) Representative fluorescence images of isolated DLD-1 (upper panel) and HCT116 (lower panel) clones stably expressing Ub-R-BC1-CB. Nuclei were stained with DAPI, scale bar: 50 µm. (**B**) Genotyping of HCT116_AAVS1_Ub-R-BC1-CB and DLD-1_AAVS1_Ub-R-BC1-CB. Genomic DNA of host cells was extracted and subjected to PCR using the primer strategy illustrated in Figure 4B. Corresponding PCR products are indicated by arrow. (**C**) Representative fluorescence images of DLD-1 cell lines stably expressing the indicated BC1-CB. Left image illustrates DLD-1_BC1-CB cells generated by random integration of the non-modified BC1-CB. The right image shows DLD-1_AAVS1_Ub-R-BC1-CB cells generated by CRISPR/Cas9-mediated site-directed integration of the ubiquitin fused BC1-CB, scale bar: 50 µm. (**D**) Quantification of nuclear CB fluorescence in DLD-1_AAVS1_Ub-R-BC1-CB cells upon compound treatment. DLD-1_AAVS1_Ub-R-BC1-CB were either treated with the indicated concentrations of FH535 or XAV939 or treated with 0.01% DMSO as control. Cells were continuously imaged every 2 h for up to 24 h. Fluorescence intensity of nuclear CB-signal was quantified and the fluorescence values were normalized to the DMSO control and plotted against time (*n* = 2, >200 cells each). Error bars: S.D.

For generation of the donor plasmid we replaced the actin binding moiety (ACT-NB) with the CTNNB1 binding nanobody BC1 using PstI and BspEI restriction site as outlined in Figure 4A and applied our CRISPR strategy as described above. Following puromycin selection we identified single clones of DLD-1 and HCT116 cells displaying a green fluorescence, which were isolated and expanded as monoclonal cell lines (Figure 6A). To confirm correct CB integration at the AAVS1 locus we performed PCR-based genotyping as illustrated in Figure 6B (strategy outlined in Figure 4B). By this we detected PCR products at ~1400 bps in DLD-1_AAVS1_Ub-R-BC1-CB and HCT116_AAVS1_Ub-R-BC1-CB cells, which were absent in the parental cells. Sequence analysis of the amplicons further verified that in both cell lines the CB transgene was successfully integrated into the AAVS1 locus. Next, we compared the phenotype of a monoclonal DLD-1 cell line generated by random integration of the non-modified BC1-CB with our newly generated DLD-1_AAVS1_Ub-R-BC1-CB cell line by fluorescence imaging (Figure 6C, Figure S2). For the DLD-1_AAVS1_Ub-R-BC1-CB cells we observed a more homogenous phenotype regarding cell area and CB fluorescence, while the conventional DLD-1_BC1-CB cells displayed a more heterogeneous morphology accompanied by miscellaneous CB signal intensities (Figure S2). Finally, we applied the novel generated DLD-1_AAVS1_Ub-R-BC1-CB cell line to monitor the effect of two compounds, FH535 and XAV939, which were previously reported to affect the level of endogenous CTNNB1 [50,51]. We performed time lapse imaging of DLD-1_AAVS1_Ub-R-BC1-CB for 24 h followed by quantitative analysis of CB fluorescence intensity in the nucleus upon treatment with different inhibitor concentrations (Figure 6D, Figure S3). By monitoring the BC1-CB fluorescence over time we observed a strong reduction of CTNNB1 to ~50% upon treatment with 10 µM and 50 µM of FH535 after 6 h. For XAV939 we detected milder effects as indicated by a reduction of the CB fluorescence to ~80% at the highest concentration of 10 µM after 12 h (Figure 6D). To verify whether the decreased CB fluorescence actually reflects a reduction of hypophosphorylated CTNNB1, we performed immunoblot analysis of DLD-1_AAVS1-Ub-R-BC1-CB cells treated either with DMSO as control or 10 µM FH535 for 24 h (Figure S4). While we observed only a minor effect of compound treatment for total CTNNB1 we observed a clear reduction of hypophosphorylated CTNNB1 in the soluble protein fraction upon treatment with FH535 (Figure S4).

Taken together, these data demonstrate that our CRISPR-based strategy to introduce optimized turnover-accelerated CB at a defined genomic locus results in the generation of highly versatile and disease relevant CB cell models. In combination with quantitative live-cell imaging these models can now be applied to monitor even subtle dose- and time-dependent compound effects on the level of endogenous proteins.

## 4. Discussion

Considering the importance of cellular imaging in biomedical research and preclinical drug development there is a continuous need for advanced labelling strategies to reliably visualize cellular components in a physiologically meaningful state [3,52]. During the last decade, fluorescently labelled nanobodies (chromobodies, CBs) emerged as versatile nanoprobes for cellular imaging of endogenous targets in living cells [9,10,12,53]. Recently, we demonstrated that quantitative analysis of CB fluorescence can additionally be employed to monitor changes in the concentration of endogenous proteins due to a mechanism termed antigen-mediated CB stabilization (AMCBS) [24]. To monitor changes in antigen concentration with high precision a strong CB expression has to be combined with a fast CB turnover [24]. However, for some CBs we previously observed that high expression levels are accompanied with the formation of intracellular aggregates. Here, we showed that expression of CBs as ubiquitin fusions can not only be used to generate turnover-accelerated CBs but also can be applied to reduce the fraction of intracellularly aggregated CBs. Our results are consistent with previous findings reporting an increased solubility and functionality of recombinant proteins expressed as ubiquitin fusions in mammalian cells and bacteria [30,54]. While the underlying mechanism is not fully understood, a chaperone-similar function is suggested, where a partially unfolded nascent protein weakly interacts with the nearby, upstream located ubiquitin moiety and thereby transiently precludes unspecific intermolecular interactions [30].

To date most stable CB cell lines are still generated by transfection of an expression plasmid followed by antibiotic selection of cells comprising a stable genomic integration of the CB transgene. However, this method is very imprecise since neither the integration site nor the number of integrated copies of the transgene can be adjusted. Additionally, the most widely used CMV promoter is prone to epigenetic silencing [35]. Accordingly, we noticed heterogeneous and overall weaker CB fluorescence intensities for a multitude of stable CB cell lines upon long-term cultivation. With the EF1-α promoter, we identified a suitable alternative promoter providing strong CB expression and stabilization ratios in the presence of the antigen which is less prone to epigenetic silencing [37]. However, with the CMV, EF1-α and h-βact promoter we compared only three different promoter types. A more comprehensive analysis of further alternatives including a determination of the methylation status of the promoter regions upon long term cultivation might reveal promoter constructs which are even better suited for a more stable CB expression.

Besides epigenetic modulation, CB expression and thus CB fluorescence is affected by the number of integrated transgenes and chromatin positioning effects [21,38]. Here, the CRISPR/Cas9 gene editing technology has been demonstrated as a highly superior approach since it enables the integration of one (heterozygous) or two (homozygous) transgene copies at a predefined genomic site [27]. In the search of transcriptionally active insertion sites, so-called genomic safe harbour sites (GSH) have been described, which allow robust and stable transgene expression. Most importantly, transgene insertion at GSH does not have adverse effect on the host cell genome. In this context, the adeno-associated virus integration site (AAVS1) on human chromosome 19 was identified as a safe genomic location for integration and high yields of transgene expression [27,42,43,44,45,46]. Based on emerging knowledge regarding CRISPR-targeted genome editing using homology-dependent repair [27,55] we used the AAVS1 locus to insert CB-based nanoprobes in human cell models. To validate the versatility and flexibility of this approach, we designed ubiquitin-fused, turnover-accelerated CB expression constructs flanked by AAVS1-specific homology arms (HA-L/R) and introduced actin- (ACT-CB) and CTNNB1-specific CB (BC1-CB) in three human cell model systems.

Although this approach is experimentally straightforward, we faced some problems, which remain to be addressed. Firstly, while the puromycin resistance should only allow the selection of clones that underwent a correct transgene insertion at the AAVS1 locus, we obtained a stable HeLa cell clone with an unspecific integration of the ACT-CB transgene. It can be speculated that due to the increased genomic instability of HeLa cells the CB transgene was inserted randomly [56]. Notably, for HCT116 and DLD-1 cells our PCR-based genotyping approach revealed only clones with a correct CB insertion at the AAVS1 integration site. Secondly, in this study CB integration was exclusively analysed by PCR-based genotyping which provides no information regarding homo- or heterozygosity of the transgene at the AAVS1 locus or possible off-target integration. This could be analysed by junction PCR using primers binding outside the homology arms or Southern blot analysis [57].

Besides targeted genomic integration and expression of a CB transgene from a GSH to visualize an endogenous antigen, gene editing can also be used to directly add a fluorescent protein (FP) to the endogenous protein of interest (PoI) [58,59,60]. However such modifications are restricted either to the N- or the C-terminus of the PoI and from our experience it is not always possible to identify suitable gRNAs to target the intended genomic loci without affecting the integrity of the endogenous protein. Additionally, as repeatedly described, FP tagging can interfere with crucial protein parameters such as turnover, subcellular localization and participation in multi-protein complexes [6,7,8].

In summary, here we combined previously established methods and conceived a strategy to generate optimized CB expressing cell lines. We illustrate that the expression of CBs as ubiquitin fusion constructs substantially increases solubility and functionality of these intracellular nanoprobes. In addition, by implementing the EF1-α promoter for stable CB expression it can be assumed that unwanted epigenetic silencing during long-term cultivation will be reduced. Lastly, we established a protocol for site-directed integration of turnover-accelerated CBs into the AAVS1 locus by using the CRISPR/Cas9 gene editing technology. By applying this approach, we engineered the AAVS1 locus of three different cell lines (DLD-1, HCT116 and HeLa cells) to stably express turnover-accelerated CBs which are suitable not only to visualize the subcellular localization and dynamics of their respective antigens but also allows the quantification of changes of the endogenous protein levels. Notably, the generated CRISPR donor CB expression vector can be easily modified for integration of different CBs or FPs.

Considering the successful demonstration we are convinced that this approach is a substantial improvement over currently applied strategies to generate stable cell lines comprising intracellularly functional nanoprobes such as CBs. To our knowledge it is the first study describing a targeted insertion of an intrabody into a GSH loci for live-cell imaging. Although not tested yet, it is conceivable that this approach is easy transferable to other live-cell imaging probes such as fluorescently labelled single chain variable fragments (scFvs) [61]. As those nanoprobes have a high tendency to aggregate, fusion to ubiquitin might be particularly beneficial. However, we have to acknowledge that several aspects such as a comprehensive analysis of other promoters for CB expression, determination of epigenetic modification of the CB transgene upon long term cell cultivation and comparative analysis of additional GSH loci are still lacking. Thus we cannot not judge whether this approach is already optimal or can be further improved. Nevertheless, we expect that our strategy will facilitate the generation of more reliable CB cell models for biomedical research and preclinical compound screening campaigns in the future using advanced cellular imaging.

## Supplementary Materials

The following are available online at http://www.mdpi.com/2073-4468/8/1/10/s1. Table S1: List of DNA oligonucleotides and synthesized gene fragments used in this study; Table S2: List of mammalian expression constructs used in this study; Figure S1: Stable HeLa_BC1-CB cell line displays heterogeneity in CB expression at high cell passages; Figure S2: Population-wide analysis of BC1-CB signal in DLD-1_BC1-CB cells and DLD-1_AAVS1_Ub-R-BC1-CB; Figure S3: Fluorescence images of DLD-1_AAVS1_Ub-R-BC1-CB cells upon compound treatment; Figure S4: FH535 reduces the amount of active CTNNB1 in DLD-1_AAVS1_Ub-R-BC1-CB cells.
