# Supplementary material for: A Strategy to Optimize the Generation of Stable Chromobody Cell Lines for Visualization and Quantification of Endogenous Proteins in Living Cells
Source: Antibodies (Basel). 2019 Jan 10;8(1):10. doi: 10.3390/antib8010010 (PMC6640688; doi:10.3390/antib8010010)
Supplement: Supplementary file 1 [file antibodies-08-00010-s001.pdf]

antibodies

MDPI

Supplementary: A strategy to optimize the generation of stable chromobody cell lines for visualization and quantification of endogenous proteins in living cells

Bettina-Maria Keller[1], *Julia Maier*[1], *Melissa Weldle*[1], *Soeren Segan*[2], *Bjoern Traenkle*[1], *Ulrich Rothbauer*[1,2*]

[1] Pharmaceutical Biotechnology, Eberhard Karls University Tuebingen, Germany

[2] Natural and Medical Sciences Institute at the University of Tuebingen, Reutlingen, Germany

Correspondence: Prof. Dr. Ulrich Rothbauer, Natural and Medical Sciences Institute at the University of Tuebingen

Markwiesenstr. 55, 72770 Reutlingen, Germany.

E-mail: ulrich.rothbauer@uni-tuebingen.de

Phone: +49 7121 51530-415

Fax: +49 7121 51530-816

Supplementary information

DNA oligo name	Sequence, 5′ - 3′
NB-ubi-for	ATA TAT CTG CAG GAG TCT GGG GGA GGC TTG GTG CA
NB-ubi-rev	ATA TAT TCC GGA GGA GAC GGT GAC CTG GGT CCC
β-actin-promoter-for	GGA ATT AAT ACT GCC TGG CCA CTC CAT G
β-actin-promoter-rev	TCC GCT AGC TCG GCA AAG GCG AGG C
β-actin-promoter-mutPstI-for	AGA GCT CCT TGT GCA GGA GCG
β-actin-promoter-mutPstI-rev	TGG AGG GCA TGG AGT GGC
AAVS1-CB-donor-fragment-2-for	TAG AGG CGG CAA TTG TTC A
AAVS1-CB-donor-fragment-2-rev	TGT TGT TAA CTT GTT TAT TGC AGC
Seq-AAVS1-CB-donor-1	TAT GGA AAA ACG CCA GCA AC
Seq-AAVS1-CB-donor-2	ATG TGG CTC TGG TTC TGG G
Seq-AAVS1-CB-donor-3	AGC GGC TCG GCT TCA C
Seq-AAVS1-CB-donor-4	CCT TAG ATG TTT TAC TAG CCA GAT
genPCR-AAVS1-int-for	TCG ACT TCC CTT CTT CCG ATG
genPCR-AAVS1-int-rev	CTC AGA TTC TGG GAG AGG GTA
EF1α-promoter (gBlock® gene fragment)	TTACCGCCATGCATTAGTTATTAATGGCTCCGGTGCCCGTCAGTGGGCAGAGCGCACAT<br>CGCCCACAGTCCCCGAGAAGTTGGGGGGAGGGGTCGGCATTTGAACCGGTGCCTAGAG<br>AAGGTGGCGCGGGGTAAACTGGGAAAGTGATGTCGTGTACTGGCTCCGCCTTTTTCCCG<br>AGGGTGGGGGAGAACCGTATATAAGTGCAGTAGTCGCCGTGAACGTTCTTTTTCGCAA<br>CGGGTTTGCCGCCAGAACACAGGTAAGTGCCGTGTGTGGTTCCCGCGGGCCTGGCCTCT<br>TTACGGGTTATGGCCCTTGCGTGCCTTGAATTACTTCCACCTGGCTCCAGTACGTGATTC<br>TTGATCCCGAGCTTCGGGTTGGAAGTGGGTGGGAGAGTTCGAGGCCTTGCGCTTAAGG<br>AGCCCCTTCGCCTCGTGCTTGAGTTGAGGCCTGGCCTGGGCGCTGGGGCCGCCGCGTGC<br>GAATCTGGTGGCACCTTCGCGCCTGTCTCGCTGCTTTCGATAAGTCTCTAGCCATTTAAA<br>ATTTTTGATGACCTGCTGCGACGCTTTTTTTCTGGCAAGATAGTCTTGTAAATGCGGGCC<br>AAGATCTGCACACTGGTATTTCGGTTTTTGGGGCCGCGGGCGGCGACGGGGCCCGTGC<br>GTCCCAGCGCACTTGTTCGGCGAGGCGGGGCCTGCGAGCGCGGCCACCGAGAATCGGA<br>CGGGGGTAGTCTCAAGCTGGCCGGCCTGCTCTGGTGCCTGGTCTCGCGCCGCCGTGTAT<br>CGCCCCGCCCTGGGCGGCAAGGCTGGCCCGGTCGGCACCAGTTGCGTGAGCGGAAAG<br>ATGGCCGCTTCCCGGCCCTGCTCCAGGGAGCTCAAAATGGAGGACGCGGCGCTCGGGA<br>GAGCGGGCGGGTGAGTCACCCACACAAAGGAAAAGGGCCTTTCCGTCCTCAGCCGTCG<br>CTTCATGTGACTCCACGGAGTACCGGGCGCCGTCCAGGCACCTCGATTAGTTCTCGAGC<br>TTTTGGAGTACGTCGTCTTTAGGTTGGGGGGAGGGGTTTTATGCGATGGAGTTTCCCCAC<br>ACTGAGTGGGTGGAGACTGAAGTTAGGCCAGCTTGGCACTTGATGTAATTCTCCTTGGA<br>ATTTGCCCTTTTTGAGTTTGGATCTTGGTTCATTCTCAAGCCTCAGACAGTGGTTCAAAG<br>TTTTTTTCTTCCATTTCAGGTGTCGTGAGCTAGCGCCACCATGCAGATCTTCG
AAVS1-CB-donor fragment 1 (gene synthesis, plasmid DNA)	CCTTTTGCTGGCCTTTTGCTCACATGTTGCTTTCTCTGACCAGCATTCTCTCCCCTGGGCC<br>TGTGCCGCTTTCTGTCTCCAGCTTGTGGCCTGGGTCACCTCTACGGCTGGCCCAGATCCT<br>TCCCTGCCGCCTCCTTCAGGTTCCGTCTTCCTCCACTCCCTCTTCCCCTTGCTCTCTGCTG<br>TGTTGCTGCCCAAGGATGCTCTTTCCGCAGCACTTCCTTCTCGGCGCTGCACCACGTGAT<br>GTCCTCTGAGCGCATCCTCCCCGTGTCTGGGTCCTCTCCGGGCATCTCTCCTCCCTCACC<br>CAACCCCATGCCGTCTTCACTCGCTGGGTTCCCTTTTCCTTCTCCTTCTGGGGCCTGTGCC<br>ATCTCTCGTTTCTTAGGATGGCCTTCTCCGACGGATGTCTCCCTTGCGTCCCGCCTCCCCT<br>TCTTGTAGGCCTGCATCATCACCGTTTTTCTGGACAACCCCAAAGTACCCCGTCTCCCTG<br>GCTTTAGCCACCTCTCCATCCTCTTGCTTTCTTTGCCTGGACACCCCGTTCTCCTGTGGAT<br>TCGGGTCACCTCTCACTCCTTTCATTTGGGCAGCTCCCCTACCCCCCTTACCTCTCTAGTC<br>TGTGCAAGCTCTTCCAGCCCCCTGTCATGGCATCTTCCAGGGGTCCGAGAGCTCAGCTA<br>GTCTTCTTCCTCCAACCCGGGCCCCTATGTCCACTTCAGGACAGCATGTTTGCTGCCTCC<br>AGGCATCCTGTGTCCCCGAGCTGGGACCACCTTATATTCCCAGGGCCGGTTAATGTGGC<br>TCTGGTTCTGGGTACTTTTATCTGTCCCCTCCACCCCACAGTGGGGCAAGCTTCTGACCT<br>CTTCTCTTCCTCCCACAGGGCCTCGAGAGATCTGGCAGCGGAGAGGGCAGAGGAAGTC<br>TTCTAACATGCGGTGACGTGGAGGAGAATCCCGGCCCTAGGCTCGAGATGACAGAATA<br>CAAACCCACGGTGCGGCTCGCGACTCGCGATGACGTGCCCCGAGCGGTGAGAACATTG<br>GCAGCAGCGTTCGCAGACTATCCGGCTACGCGGCATACTGTCGATCCTGATCGACATAT<br>TGAACGGGTCACCGAGTTGCAGGAGCTTTTCCTCACACGCGTTGGATTGGATATTGGCA<br>AAGTATGGGTCGCGGACGACGGGGCAGCTGTCGCCGTGTGGACCACCCCCGAGTCAGT<br>GGAGGCTGGAGCGGTATTCGCTGAGATCGGCCCTCGGATGGCAGAATTGAGCGGCTCC<br>AGACTGGCGGCTCAACAGCAGATGGAAGGGCTCCTCGCCCCTCATAGACCTAAGGAAC<br>CTGCTTGGTTCCTCGCAACCGTGGGCGTCTCACCAGACCATCAGGGGAAGGGCCTTGG<br>GTCTGCGGTGGTCTTGCCGGGGGTCGAGGCaGCAGAGAGAGCTGGGGTACCCGCGTTTT<br>TGGAAACAAGTGCGCCCCGAAACCTCCCGTTTTACGAACGGCTTGGCTTTACAGTCACA

	GCAGATGTTGAAGTACCGGAGGGACCAAGGACCTGGTGCATGACCCGCAAGCCGGGA<br>GCTTGATCAAGAGGGCCCGTTTAAACCCGCTGATCAGCCTCGACTGTGCCTTCTAGTTG<br>CCAGCCATCTGTTGTTTGCCCCTCCCCCGTGCCTTCCTTGACCCTGGAAGGTGCCACTCC<br>CACTGTCCTTTCCTAATAAAATGAGGAAATTGCATCGCATTGTCTGAGTAGGTGTCATT<br>CTATTCTGGGGGGTGGGGTGGGGCAGGACAGCAAGGGGGAGGATTGGGAAGACAATA<br>GCAGGCATGCTGGGGATGCGGTGGGCTCTATGGACTAGTATTAATTAAATCTAGAGGC<br>GGCAATTGTTCACTCCTCAGGTGCAGGC
AAVS1-CB-donor fragment 2 (gBlock® gene fragment)	AAATCTAGAGGCGGCAATTGTTCACTCCTCAGGTGCAGGCTGCCTATCAGAAGGTGGT<br>GGCTGGTGTGGCCAATGCCCTGGCTCACAAATACCACTGAGATCTTTTTCCCTCTGCCA<br>AAAATTATGGGGACATCATGAAGCCCCTTGAGCATCTGACTTCTGGCTAATAAAGGAA<br>ATTTATTTTCATTGCAATAGTGTGTTGGAATTTTTTGTGTCTCTCACTCGGAAGGACATAT<br>GGGAGGGCAAATCATTTAAAACATCAGAATGAGTATTTGGTTTAGAGTTTGGCAACAT<br>ATGCCATATGCTGGCTGCCATGAACAAAGGTGGCTATAAAGAGGTCATCAGTATATGA<br>AACAGCCCCCTGCTGTCCATTCCTTATTCCATAGAAAAGCCTTGACTTGAGGTTAGATTT<br>TTTTTATATTTTGTTTTGTGTTATTTTTTTCTTTAACATCCCTAAAATTTTCCTTAGATGTTT<br>TACTAGCCAGATTTTTCCTCCTCTCCTGACTACTCCCAGTCATAGCTGTCCCTCTTCTCGT<br>CGACAGTACTAAGCTTTGACAGAAAAGCCCCATCCTTAGGCCTCCTCCTTCCTAGTCTC<br>CTGATATTGGGTCTAACCCCCACCTCCTGTTAGGCAGATTCCTTATCTGGTGACACACCC<br>CCATTTCCTGGAGCCATCTCTCTCCTTGCCAGAACCTCTAAGGTTTGCTTACGATGGAGC<br>CAGAGAGCATCCTGGGAGGGAGAGCTTGGCAGGGGGTGGGAGGGAAGGGGGGGATG<br>CGTGACCTGCCCGGTTCTCAGTGGCCACCCTGCGCTACCCTCTCCCAGAACCTGAGCTG<br>CTCTGACGCGGCTGTCTGGTGCGTTTCACTGATCCTGGTGCTCCAGCTTCCTTACACTTC<br>CCAAGAGGAGAAGCAGTTTGGAAAAACAAAATCAGAATAAGTTGGTCCTGAGTTCTA<br>ACTTTGGCTCTTCACCTTTCTAGTCCCCAATTTATATTGTTCCTCCGTGCGTCAGTTTTAC<br>CTGTGAGATAAGGCCAGTAGCCAGCCCCGTCCTGGCAGGGCTGTGGTGAGGAGGGGG<br>GTGTCCGTGTGGAAAACTCCCTTTGTGAGAATGGTGCGTCCTAGGTGTTCACCAGGTCG<br>TGGCCGCCTCTACTCCCTTTCTCTTTCTCCATCCTTCTTTCCTTAAAGAGTCCCCAGTGCT<br>ATCTGGGACATATTCCTCCGCCCAGAGCAGGGTCCCGCTTCCCTAAGGCCCTGCTCTGG<br>GCTTCTGGGTTTGAGTCCTTGGCAAGCCCAGGAGAGGCGCTCAGGCTTCCCTGTCCCCC<br>TTCCTCGTCCACCATCTCATGCCCCTGGCTCTCCTGCCCCTTCCCTACAGGGGTTCCTGG<br>CTCTGCTCTCAATTGTTGTTGTTAACTTGTTTATTGCAGCTTATAATGG

Table S1: List of DNA oligonucleotides and synthesized gene fragments used in this study

	Expression construct
1.	pEGFP-N1 (Clontech)
2.	Ub-M-BC1-CB [1]
3.	Ub-R-BC1-CB [1]
4.	LMN-CB [2]
5.	Ub-M-LMN-CB (this study)
6.	Ub-R-LMN-CB (this study)
7.	(EF1α)-Ub-R-BC1-CB (this study)
8.	(β-actin)-Ub-R-BC1-CB (this study)
9.	mCherry [3]
10.	mCherry-CTNNB1 [1]
11.	Cas9_gRNA [4]
12.	AAVS1_Ub-R-BC1-CB (this study)
13.	Actin-CB [5]
14.	AAVS1_Ub-R-ACT-CB (this study)

Table S2: List of mammalian expression constructs used in this study

Supplementary Figures

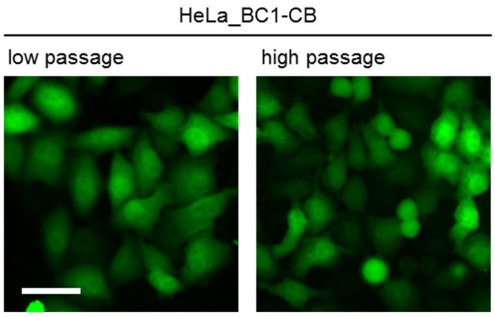

Figure S1 Stable HeLa_BC1-CB cell line displays heterogeneity in CB expression at high cell passages.

Illustration of HeLa cells stably expressing the BC1-CB driven by the CMV promoter at an early passage (passage number 4, left side) and at a high passage (passage number 20, right side). Scale bar: 50 µm.

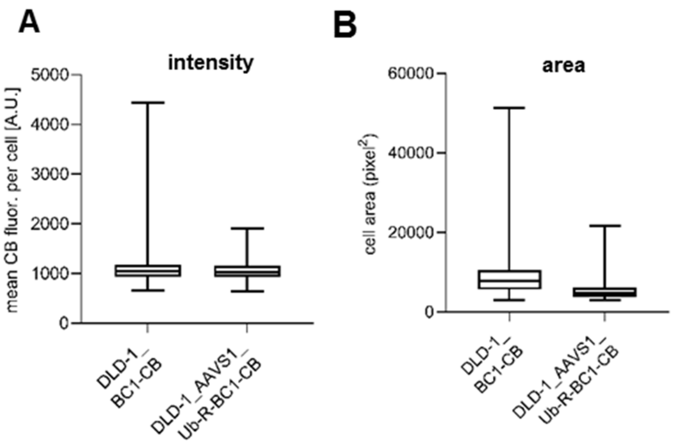

Figure S2 Population-wide analysis of BC1-CB signal in DLD-1_BC1-CB cells and DLD-1_AAVS1_Ub-R-BC1-CB.

Fluorescence images of DLD-1_BC1-CB cells and DLD-1_AAVS1_Ub-R-BC1-CB (as illustrated in **Figure 6C**) were subjected to quantitative image analysis. Using a customized image segmentation algorithm (see Material and Methods), the mean fluorescence intensity of the CB (**A**) and the cell area (**B**) were determined for each individual cell imaged. Number of analyzed cells: DLD-1_BC1: n=2478; DLD1_AAVS1_Ub-R-BC1-CB: n=2090.

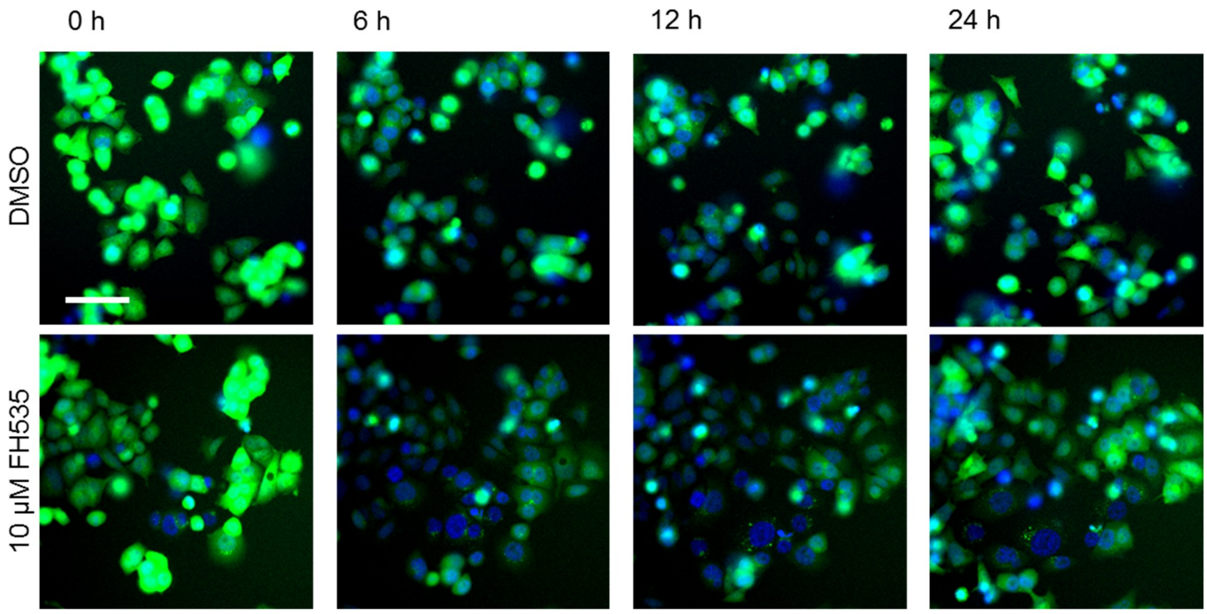

Figure S3 Fluorescence images of DLD-1_AAVS1_Ub-R-BC1-CB cells upon compound treatment.

DLD-1_AAVS1_Ub-R-BC1-CB were either treated with 10 µM FH535 or 0.01 % DMSO as control and were continuously imaged every 2 h for up to 24 h. Representative fluorescence images are shown after 0, 6, 12 and 24 h, scale bar: 50 µM.

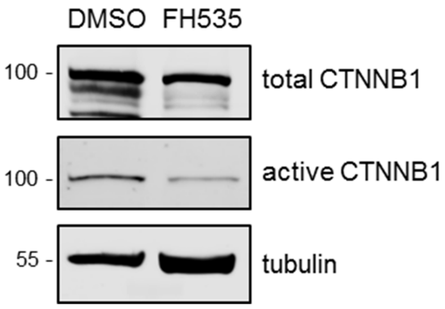

Figure S4: FH535 reduces the amount of active CTNNB1 in DLD-1_AAVS1_Ub-R-BC1-CB cells.

DLD-1_AAVS1_Ub-R-BC1-CB were either treated with 10 µM FH535 or 0.01 % DMSO as control for 24 h. Cells were lysed using lysis buffer comprising 0.5% NP-40 and equal protein amounts of the soluble fraction were subjected to SDS-PAGE followed by immunoblot analysis using antibodies specific for total CTNNB1, active CTNNB1 and tubulin as loading control.

References

1. Keller, B.M.; Maier, J.; Secker, K.A.; Egetemaier, S.M.; Parfyonova, Y.; Rothbauer, U.; Traenkle, B. Chromobodies to quantify changes of endogenous protein concentration in living cells. *Molecular & cellular proteomics : MCP* **2018**.
2. Zolghadr, K.; Gregor, J.; Leonhardt, H.; Rothbauer, U. Case study on live cell apoptosis-assay using lamin-chromobody cell-lines for high-content analysis. *Methods in molecular biology* **2012**, *911*, 569-575.
3. Toulany, M.; Maier, J.; Iida, M.; Rebholz, S.; Holler, M.; Grottke, A.; Juker, M.; Wheeler, D.L.; Rothbauer, U.; Rodemann, H.P. Akt1 and akt3 but not akt2 through interaction with DNA-pkcs stimulate proliferation and post-irradiation cell survival of k-ras-mutated cancer cells. *Cell death discovery* **2017**, *3*, 17072.
4. Oceguera-Yanez, F.; Kim, S.I.; Matsumoto, T.; Tan, G.W.; Xiang, L.; Hatani, T.; Kondo, T.; Ikeya, M.; Yoshida, Y.; Inoue, H., *et al.* Engineering the aavs1 locus for consistent and scalable transgene expression in human ipscs and their differentiated derivatives. *Methods* **2016**, *101*, 43-55.
5. Panza, P.; Maier, J.; Schmees, C.; Rothbauer, U.; Sollner, C. Live imaging of endogenous protein dynamics in zebrafish using chromobodies. *Development* **2015**, *142*, 1879-1884.

© 2019 by the authors. Submitted for possible open access publication under the terms and conditions of the Creative Commons Attribution (CC BY) license (http://creativecommons.org/licenses/by/4.0/).
